# Supplementary material for: Nuclear Magnetic Resonance Therapy Modulates the miRNA Profile in Human Primary OA Chondrocytes and Antagonizes Inflammation in Tc28/2a Cells
Source: Int J Mol Sci. 2021 May 31;22(11):5959. doi: 10.3390/ijms22115959 (PMC8198628; doi:10.3390/ijms22115959)
Supplement: Supplementary file 1 [file ijms-22-05959-s001.zip › ijms-1204205 Supplementary.pdf]

Supplementary data

Article

Nuclear Magnetic Resonance Therapy Modulates the miRNA Profile in Human Primary OA Chondrocytes and Antagonizes Inflammation in Tc28/2a Cells

Bibiane Steinecker-Frohnwieser [1], Birgit Lohberger [1,2,*], Nicole Eck [1,2], Anda Mann [3], Cornelia Kratschmann [3], Andreas Leithner [2], Werner Kullich [1] and Lukas Weigl [3]

[1] Ludwig Boltzmann Institute for Arthritis and Rehabilitation, 5760 Saalfelden, Austria; bibiane.frohnwieser@medunigraz.at (B.S.-F.); nicole.eck@medunigraz.at (N.E.); lbirehab@aon.at (W.K.)

[2] Department of Orthopedics and Trauma, Medical University Graz, 8036 Graz, Austria; andreas.leithner@medunigraz.at

[3] Division of Special Anaesthesia and Pain Medicine, Medical University of Vienna, 1090 Vienna, Austria; anda.mann@meduniwien.ac.at (A.M.); cornelia.kratschmann@meduniwien.ac.at (C.K.); lukas.weigl@meduniwien.ac.at (L.W.)

* Correspondence: birgit.lohberger@medunigraz.at

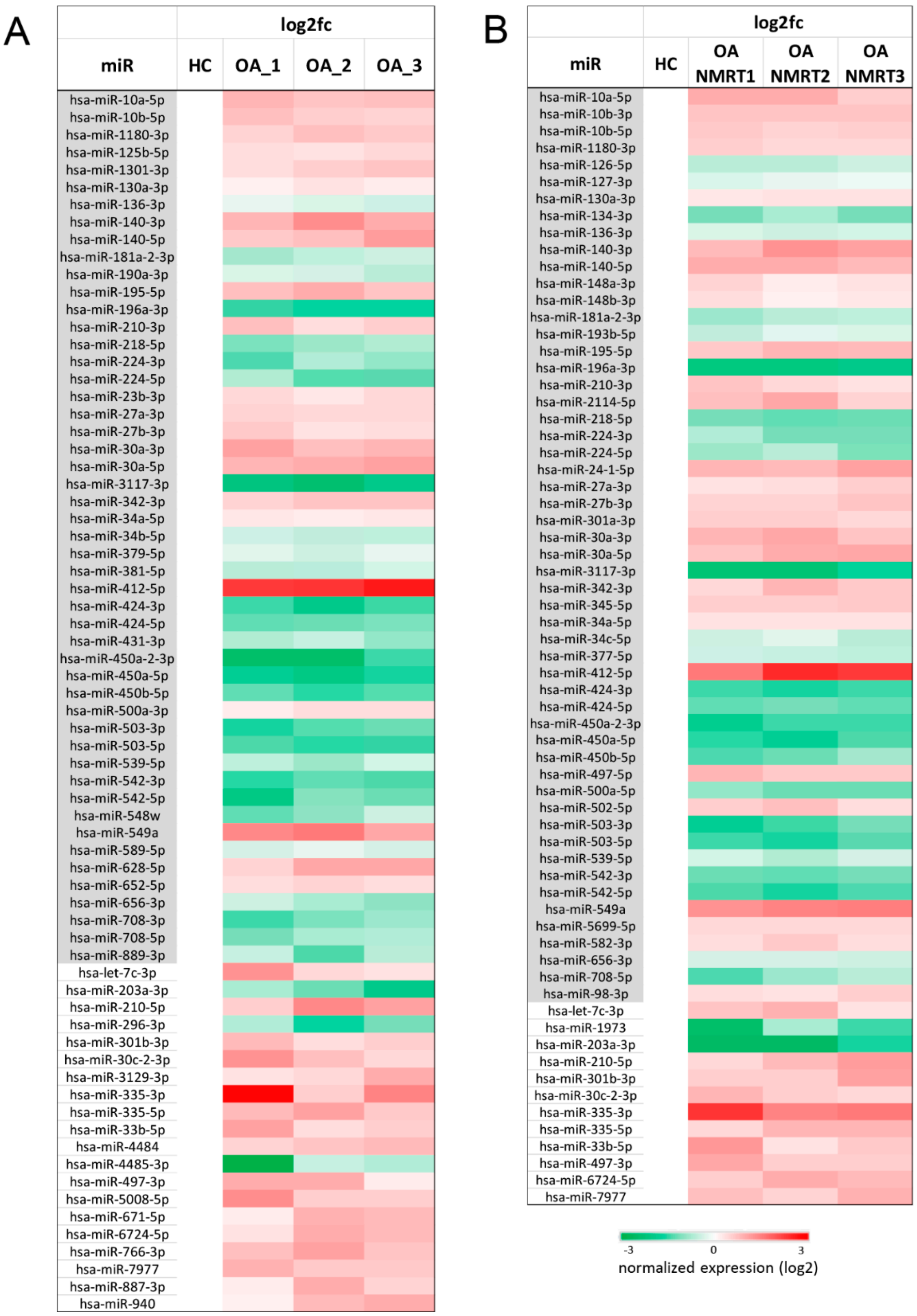

Figure S1. The log2 fold change between (**A**) HC vs. OA cells and (**B**) vs. OA cells plus NMRT treated are given in form of heat maps.

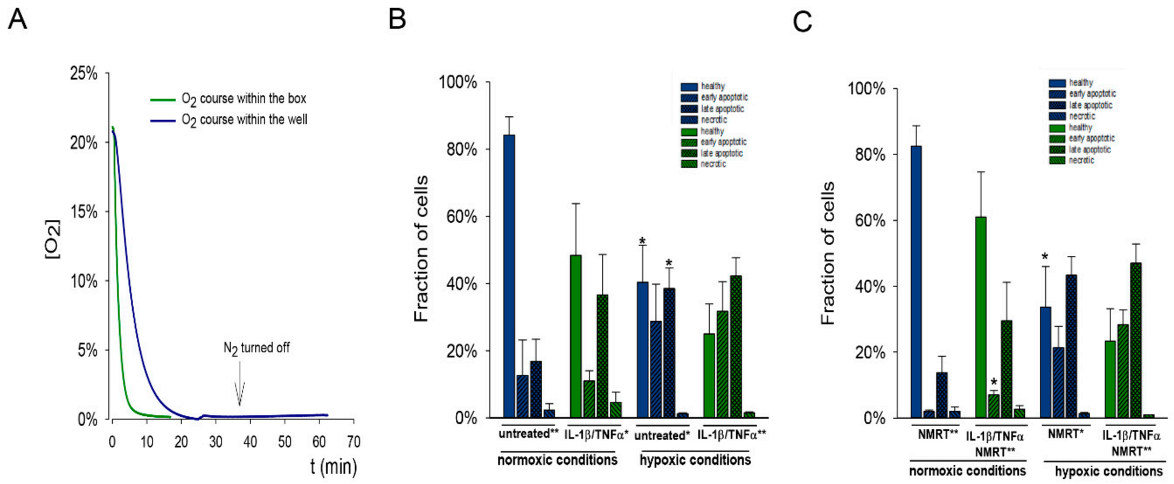

Figure S2. T/C-28a2 grown under hypoxic conditions and changes in gene expression. (A) showed a representative time course of the O_2 concentration within the air tight hypoxic chamber and the well of a cell culture plate during O_2 depletion. (B, C) The induction of apoptosis by hypoxic conditions under control conditions, IL-1β/TNFα stimulation, and NMRT treatment were measured using annexin V/PI flow cytometry. The asterisk in the x-axis text describe the differences between the single fractions per group by ANOVA (*).

	HC vs HC-OA	HC vs HC-OA+NMRT		**mechanism & targets**
hsa-miR-106a-5p			31%	Inhibits proliferation and migration in astrocytes, promotes apoptosis [S1].
**hsa-miR-122-5p**			32%	Targets IGF1R in breast cancer cells [S2]; correlates with disease severity of knee OA [S3].
hsa-miR-1249-3p			57%	Inhibits tumor growth, metastasis, and angiogenesis by targeting VEGFA and HMGA2 [S4].
hsa-miR-155-3p			43%	Regulates inflammation and autophagy by targeting SHIP1, FoxO3, Ulk1, Atg14, Atg5 in RA [S5].
**hsa-miR-203a-3p**			-47%	Down-regulation suppresses IL-1β induced effects in chondrocytes through Smad3 [S6].
**hsa-miR-24-1-5p**			32%	Promotes cell proliferation and inhibits chondrocyte apoptosis in rats via *c-Myc* and downregulation of MAPK signaling [S7].
hsa-miR-326			63%	Function as a tumor suppressor [S8].
hsa-miR-3613-3p			-31%	Affects cell proliferation and cell cycle in hepatocellular carcinoma [S9].
**hsa-miR-365a-5p**			-37%	Regulates chondrocyte differentiation via targeting HDAC4 [S10] and prevents IL-1β-stimulated loss of ECM and MMP-13 expression [S11].
**hsa-miR-4284**			30%	Regulates articular cartilage homeostasis by altering chondrocyte metabolism, targets TRAF4, HDAC, PTEN [S12].
hsa-miR-450a-2-3p			38%	Targets STAT1 and triggers osteoblastic differentiation of hMSCs [S13].
hsa-miR-500a-5p			-50%	Regulates oxidative stress response in breast cancer cells [S14].
hsa-miR-502-3p			42%	Suppresses cell proliferation in cancer cells [S15].
**hsa-miR-502-5p**			100%	Inhibits IL-1β-induced chondrocyte injury by targeting TRAF2 [S16].
hsa-miR-539-3p			35%	Acts as tumor suppresor [S17].
hsa-miR-576-5p			33%	Targets TFAP2A and elevates cell proliferation and invasion of human trophoblasts [S18].
hsa-miR-668-3p			57%	Targets SDF-1/CXCR4 signaling in rat H9c2 cardiomyocyte [S19].
hsa-miR-769-3p			100%	Down-regulates NDRG1 and enhances apoptosis in MCF-7 cells during reoxygenation [S20].
hsa-miR-889-3p			33%	Tumor suppressor by targeting FGFR2 [S21] and promotes proliferation of osteosarcoma by inhibiting MNDA expression [S22].

Table S1. MiRs characterized by a 30% difference in the fold change of HC vs OC cells on one site and HC vs OA cells plus NMRT on the other site are listed. Downregulations (green arrow downstream), upregulations (red arrow upstream), and unchanged expressions (blue equivalent arrows) are given. The extent of the changes as a percentage and physiologic relevant effects are listed; miRs playing a role in chondrocyte physiology are given in bold.

Primer Sequences Used for Real-Time PCR				
	Oligonucleotide sequences			
Target gene	forward Primer	reverse Primer	Acc. Nr	amplicon size (bp)
COX2	GGCTTCCATTGACCAGAGCAG	GCCGAGGCTTTTCTACCAGA	M90100.1	194
EGF	AAGGTACTCTCGCAGGAAATGG	ACATACTCTCTCTTGCCTTGACC	NM_001963	248
βFGF2	AGAAGAGCGACCCTCACATCA	ACTGCCCAGTTCGTTTCAGTG	NM_002006.5	237
HDAC4	CTGGCTCATGAGACCTTGCC	ATCCACCGTGCTGGGCAT	NM_001378414	270
IGF2	CCTCCAGTTCGTCTGTGGG	CACGTCCCTCTCGGACTT	NM_000576	186
IGFBP3	GCCAGCTCCAGGAAATGCTA	GGGGTGGAACTTGGGATCAG	NM_000598	109
IL-1β	AGCTACGAATCTCCGACCAC	CGTTATCCCATGTGTCGAAGAA	NM_000576.2	186
iNOS	TTCAGTATCACAACCTCAGCAAG	TGGACCTGCAAGTTAAAATCCC	BC144126	207
MMP13	TCCTCTTCTTGAGCTGGACTCATT	CGCTCTGCAAACTGGAGGTC	NM_002427.4	97
MMP3	TTTTGGCCATCTCTTCCTTCA	TGTGGATGCCTCTTGGGTATC	NM_002422.5	139
PDGFα	CCAGCGACTCCTGGAGATAGA	CGTCCTGGTCTTGCAGACAG	NM_002607.5	169
RUNX2	TCCTATGACCAGTCTTACCCCT	GGCTCTTCTTACTGAGAGTGGAA	AF001450	190
TGFβ	CAAGCAGAGTACACACAGCAT	TGCTCCACTTTTAACTTGAGCC	NM_000660	129
TNFα	ATGAGCACTGAAAGCATGATCC	GTCACTCGGGGTTCGAGAAG	NM_000594	258
VEGFA	CGCAGCTACTGCCATCCAAT	GTGAGGTTTGATCCGCATAATCT	NM_001025366.3	192
HIF1	Hs_HIF1A_1_QuantiTect Primer Assay	QT00083664	NM_001243084	104
HIF2	Hs_EPAS1_1_QuantiTect Primer Assay	QT00069587	NM_001430	127
GAPDH	TGATGACATCAAGAAGGTGGTGAAG	TCCTTGGAGGCCATGTGGGCCAT	NM_002046	102
Aldolase	ATGAGTCCACTGGGAGCATTG	ACCGCCCTTGGATTTGATAAC	NM_000034	209
ETIF3	CTACCAGCCGTTCAGCAAAG	CACCACCAAACTGAGAGGAGT	NM_003753	110

Table S2. Primer sequences used for qPCRs are given. Target gene and nucleotide-sequences in 5′-3′ direction for the forward and reverse primer as well as the accession numbers and amplicon sizes are listed.

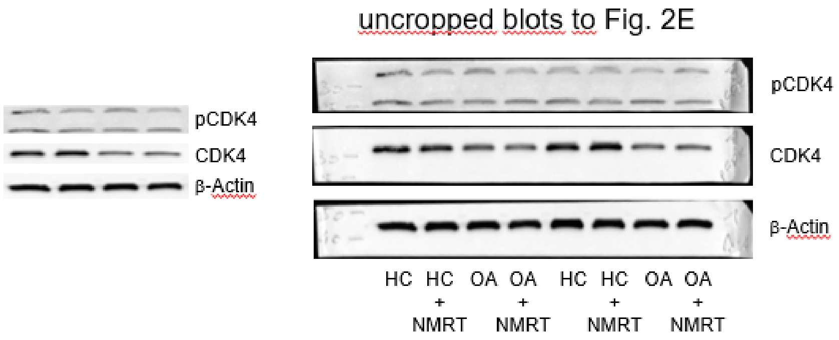

Uncropped blots to Figure 2E. The original blots show two different OA primary cultures.

Additional references (supplementary data)

S1. Zhi, F.; Zhou, G.; Shao, N.; Xia, X.; Shi, Y.; Wang, Q.; Zhang, Y.; Wang, R.; Xue, L.; Wang, S.; Wu, S.; Peng, Y.; Yang, Y. miR-106a-5p Inhibits the Proliferation and Migration of Astrocytoma Cells and Promotes Apoptosis by Targeting FASTK. *PLoS One* **2013**, 8(8), e72390.

S2. Wang, B.; Wang, H.; Yang, Z. MiR-122 Inhibits Cell Proliferation and Tumorigenesis of Breast Cancer by Targeting IGF1R. *PLoS One* **2012**, 7(10), e47053.

S3. Kong, R.; Gao, J.; Si, Y.; Zhao, D. Combination of circulating miR-19b-3p, miR-122-5p and miR-486-5p expressions correlates with risk and disease severity of knee osteoarthritis. *Am J Transl Res* **2017**, 9(6), 2852-64.

S4. Chen, X.; Zeng, K.;, Xu, M.; Liu, X.; Hu, X.; Xu, T.; He, B.; Pan, Y.; Sun, H.; Wang, S. P53-induced miR-1249 Inhibits Tumor Growth, Metastasis, and Angiogenesis by Targeting VEGFA and HMGA2. *Cell Death Dis* **2019**, 10(2), 131.

S5. Kurowska-Stolarska, M.; Alivernini, S.; Ballantine, L.E.; Asquith, D.L.; Millar, N.L.; Gilchrist, D.S.; Reilly, J.; Ierna, M.; Fraser, A.R.; Stolarski, B.; McSharry, C.; Hueber, A.J.; Baxter, D.; Hunter, J.; Gay, S.; Liew, F.Y.; McInnes, I.B. MicroRNA-155 as a Proinflammatory Regulator in Clinical and Experimental Arthritis. *Proc Natl Acad Sci U S A* **2011**, 108(27), 11193-98.

S6. An, Y.; Wan, G.; Tao, J.; Cui, M.; Zhou, Q.; Hou, W. Down-regulation of microRNA-203a Suppresses IL-1β-induced Inflammation and Cartilage Degradation in Human Chondrocytes Through Smad3 Signaling. *Biosci Rep* **2020**, 40(3), BSR20192723.

S7. Wu, J.H.; Liu, W.; Zhang, L.; Liu, X.Y.; Wang, Y.; Xue, B.; Liu, B.; Duan, R.; Zhang, B.; Ji, Y. Effects of microRNA-24 Targeting C-myc on Apoptosis, Proliferation, and Cytokine Expressions in Chondrocytes of Rats with Osteoarthritis via MAPK Signaling Pathway. *J Cell Biochem* **2018**, 119(10), 7944-58.

S8. Liang, X.; Li, Z.; Men, Q.; Li, Y.; Li, H.; Chong, T. miR-326 Functions as a Tumor Suppressor in Human Prostatic Carcinoma by Targeting Mucin1. *Biomed Pharmacother* **2018**, 108, 574-83.

S9. Zhang, D.; Liu, E.; Kang, J.; Yang, X.; Liu, H. MiR-3613-3p Affects Cell Proliferation and Cell Cycle in Hepatocellular Carcinoma. *Oncotarget* **2017**, 8(54), 93014-28.

S10. Guan, Y.J.; Yang, X.; Well, L.; Chen, Q. MiR-365: A Mechanosensitive microRNA Stimulates Chondrocyte Differentiation Through Targeting Histone Deacetylase 4. *FASEB J* **2011**, 25(12), 4457-66.

S11. Hwang, H.S.; Park, S.J.; Lee, M.H.; Kim, H.A. MicroRNA-365 Regulates IL-1β-induced Catabolic Factor Expression by Targeting HIF-2α in Primary Chondrocytes. *Sci Rep* **2017**, 7(1), 17889.

S12. Tamaddon, G.; Geramizadeh, B.; Karimi, M.H.; Mowla, S.J.; Abroun, S. miR-4284 and miR-4484 as Putative Biomarkers for Diffuse Large B-Cell Lymphoma. *Iran J Med Sci* **2016**, 41(4), 334-339.

S13. Dernowsek, J.A.; Pereira, M.C.; Fornari, T.A.; Macedo, C.; Assis, A.F.; Donate, P.B.; Bombonato-Prado, K.F.; Passos-Bueno, M.R.; Passos, G.A. Posttranscriptional Interaction Between miR-450a-5p and miR-28-5p and STAT1 mRNA Triggers Osteoblastic Differentiation of Human Mesenchymal Stem Cells. *J Cell Biochem* **2017**, 118(11), 4045-4062.

S14. Esposti, D.D.; Aushev, V.N.; Lee, E.; Cros, M.P.; Zhu, J.; Herceg, Z.; Chen, J.; Hernandez-Vargas, H. miR-500a-5p Regulates Oxidative Stress Response Genes in Breast Cancer and Predicts Cancer Survival. *Sci Rep* **2017**, 7(1), 15966.

S15. Jin, H.; Yu, M.; Lin, Y.; Hou, B.; Wu, Z.; Li, Z.; Sun; J. MiR-502-3P suppresses cell proliferation, migration, and invasion in hepatocellular carcinoma by targeting SET. *Onco Targets Ther* **2016**, 9, 3281-9.

S16. Zhang, G.; Sun, Y.; Wang, Y.; Liu, R.; Bao, Y.; Li, Q. MiR-502-5p Inhibits IL-1β-induced Chondrocyte Injury by Targeting TRAF2. *Cell Immunol* **2016**, 302, 50-57.

S17. Gua, J.; Gong, G.; Zhang, B. miR-539 Acts as a Tumor Suppressor by Targeting Epidermal Growth Factor Receptor in Breast Cancer. *Sci Rep* **2018**, 8(1), 2073.

S18. Wang, X.; Peng, S.; Cui, K.; Hou, F.; Ding, J.; Wang, M. MicroRNA-576-5p Enhances the Invasion Ability of Trophoblast Cells in Preeclampsia by Targeting TFAP2A. *Mol Genet Genomic Med* **2020**, 8(1):e1025.

S19. Gao, Z.; Gao, Q.; Ly, X. MicroRNA-668-3p Protects Against Oxygen-Glucose Deprivation in a Rat H9c2 Cardiomyocyte Model of Ischemia-Reperfusion Injury by Targeting the Stromal Cell-Derived Factor-1 (SDF-1)/CXCR4 Signaling Pathway. *Med Sci Moni*t **2020**, 26, e919601.

S20. Luo, E.C.; Chang, Y.C.; Sher, Y.P.; Huang, W.Y.; Chuang, L.L.; Chiu, Y.C.; Tsai, M.H.; Chuang, E.Y. MicroRNA-769-3p Down-Regulates NDRG1 and Enhances Apoptosis in MCF-7 Cells During Reoxygenation. *Sci Rep* **2014**, 4, 5908.

S21. Sun, Y.; Cheng, Y.; Zhang, Y., HAN; K: MicroRNA-889-3p Targets FGFR2 to Inhibit Cervical Cancer Cell Viability and Invasion. *Exp Ther Med* **2019**; 18(2); 1440-1448.

S22. Ge; D.; Chen, H.; Zheng, S.; Zhang, B.; Ge, Y.; Yang, L.; Cao, X. Hsa-miR-889-3p Promotes the Proliferation of Osteosarcoma Through Inhibiting Myeloid Cell Nuclear Differentiation Antigen Expression. *Biomed Pharmacother* **2019**, 114, 108819.
